# Anton's syndrome due to cerebrovascular disease: a case report

**DOI:** 10.4076/1752-1947-3-9028

**Published:** 2009-09-09

**Authors:** Mohana Maddula, Stuart Lutton, Breffni Keegan

**Affiliations:** 1 Stroke Unit, Erne Hospital, Cornagrade Road, Enniskillen BT74 6AY, UK

## Abstract

**Introduction:**

Anton's syndrome describes the condition in which patients deny their blindness despite objective evidence of visual loss, and moreover confabulate to support their stance. It is a rare extension of cortical blindness in which, in addition to the injury to the occipital cortex, other cortical centres are also affected, with patients typically behaving as if they were sighted.

**Case presentation:**

We present a case report of an 83-year-old white woman with cortical blindness as a result of bilateral occipital lobe infarcts. Despite her obvious blindness, illustrated by her walking into objects, the patient expressed denial of visual loss and demonstrated confabulation in her accounts of her surroundings, consistent with a diagnosis of Anton's syndrome.

**Conclusions:**

A suspicion of cortical blindness and Anton's syndrome should be considered in patients with atypical visual loss and evidence of occipital lobe injury. Cerebrovascular disease is the most common cause of Anton's syndrome, as in our patient. However, any condition that may result in cortical blindness can potentially lead to Anton's syndrome. Recovery of visual function will depend on the underlying aetiology, with cases due to occipital lobe infarction after cerebrovascular events being less likely to result in complete recovery. Management in these circumstances should accordingly focus on secondary prevention and rehabilitation.

## Introduction

Visual anosognosia, that is, denial of loss of vision, associated with confabulation in the setting of obvious visual loss and cortical blindness is known as Anton's syndrome. Although the anterior visual tracts are intact, the visual association centres in the occipital cortex may be compromised. Patients with Anton's syndrome strongly believe they can see what they cannot and behave and talk as though they were sighted. Attention to the possibility of the condition is, however, drawn when they walk into walls, fall over furniture and describe objects that are not present. We describe a case of a patient with Anton's syndrome and its associated features.

## Case presentation

An 83-year-old white woman with a background of mild dementia, hypothyroidism and a resected gastric carcinoma 20 years earlier was found collapsed on the floor of her house. It was difficult to exclude loss of consciousness reliably, but when she was seen by her GP a right hemiparesis was reported, which had resolved by the time of admission to hospital. Her premorbid functional capacity had been good, and she could mobilise independently with the aid of a Zimmer frame and had required minimal assistance with activities of daily living.

On arrival to the stroke unit, the patient's Glasgow Coma Scale score (GCS) was 15 out of 15 and she had normal power in all four limbs and no sensory loss. Her most striking clinical feature on examination was severe impairment of visual acuity. She was walking into objects and was clearly blind. Despite an objective diminution of her vision, our patient maintained she was able to 'see' things around her. Pupillary reflexes were intact (suggesting an intact anterior visual pathway), with fundoscopy unremarkable. The only other neurological finding was a mild receptive dysphasia.

**Figure 1:**
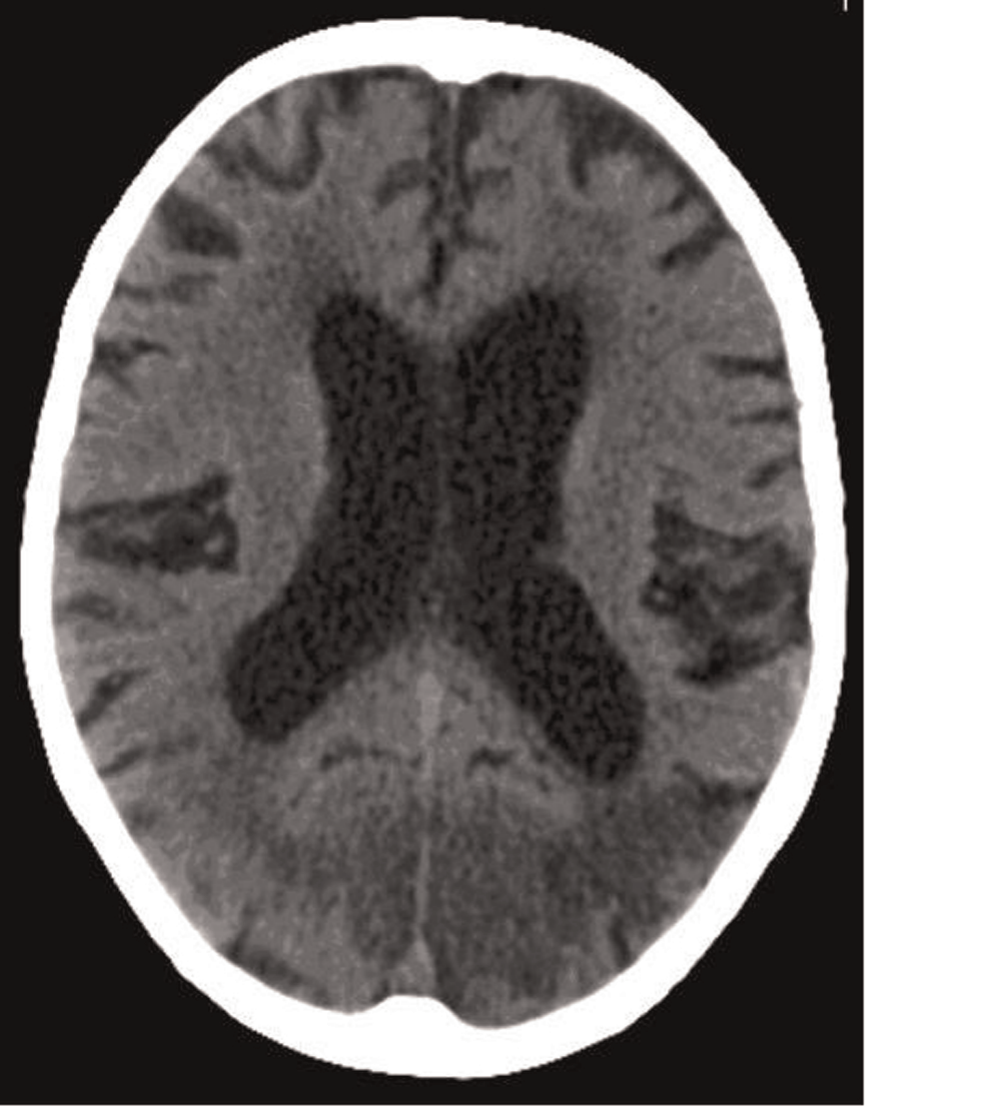
**A computed tomography scan of the patient's brain at initial presentation, demonstrating acute infarction in the right occipital and left occipito-parietal lobes**.

A computed tomography scan of the patient's brain (Figure 1) demonstrated evidence of acute infarction in the right occipital and left occipito-parietal lobes, on a background of generalised periventricular ischaemia, consistent with a diagnosis of cortical blindness. Her receptive dysphasia resolved early in the course of her hospitalisation, but during her rehabilitiation she nonetheless continued to deny any loss of vision and showed signs of confabulation. When asked to comment on the doctor's tie, she was quick with an answer, but one that was incorrect. Interestingly, towards the end of her admission, she asked a nurse to "light up some candles" because she felt the room was dark, suggesting a degree of light perception that was not present on admission. She required assistance in mobilising safely in view of her visual impairment, and required help for most activities of daily living. Although she was, for example, unable to see her meals, she would feel for the utensils on the tray when it was placed in front of her, and if left to her own devices she would start eating the food, but assistance was required to help her finish the meals and avoid spillage. Although she would deny visual deficit, she would accept such assistance.

Subsequently, she returned home to live with a family member, but she required carers visiting regularly to assist in activities of daily living such as washing and dressing. Her general condition, such as nutritional status and physical strength, improved. About 4 weeks after initial presentation it was reported by our patient's family that they believed she was now able to follow shadows, although this may have been due to 'blindsight'. However, she still required significant carer involvement some months later. Although there had perhaps been a slight improvement, the likelihood of a marked recovery in visual acuity remained low.

## Discussion

The French renaissance writer Montaigne (1533-1592) described in his second book of *Les Essais* the case of a nobleman who did not believe he was blind despite the obvious signs [1]. This was probably the first ever description of not perceiving one's own blindness in the absence of psychiatric illness or underlying cognitive impairment.

A few hundred years later the Austrian neuropsychiatrist Gabriel Anton (1858-1933) described patients with objective blindness and deafness who showed a lack of self-perception of their deficits. He associated these with brain pathology [2]. Joseph François Babinski (1857-1932) later used the term anosognosia to describe this phenomenon [3].

Neurological visual impairment, in which the visual disturbance is as a result of brain abnormality or damage rather than eye abnormalities, encompasses a broad spectrum of conditions. These include conditions such as cerebral visual impairment, visual neglect, visual agnosia, various visual perceptual disorders, homonymous hemianopia, lack of facial recognition, delayed visual development and cortical blindness.

In patients with total cortical blindness secondary to bilateral damage to the occipital cortices, movement of objects may nonetheless be perceived, either consciously [4] (Riddoch's syndrome) or unconsciously (blindsight) [5]. Conversely, motion blindness, in which patients can see objects but cannot perceive the motion of these objects, has also been well described [6]. This may be accounted for by the presence of projections from the lateral geniculate nucleus, both to the visual cortex (V1) via the optic radiations and to the motion-selective middle temporal area (MT or V5), a cortical area not previously considered 'primary' [7]. Other manifestations of impaired visual acuity may include Charles Bonnet syndrome, in which patients with visual loss from any cause may experience hallucinations, often very elaborate, with images of unfamiliar people or buildings, and so on, although with preservation of insight [8].

Anton's syndrome is the denial of loss of vision (visual anosognosia) associated with confabulation in the setting of obvious visual loss and cortical blindness. Frequently, patients with damage to the occipital lobes bilaterally also have damage to their visual association cortex, which may account for their lack of awareness [9]. Additionally, as suggested by Anton, damaged visual areas are effectively disconnected from functioning areas, such as speech-language areas. In the absence of input, functioning speech areas often confabulate a response [10].

In addition to the hypothesised disconnection described above, two other likely neuropsychological mechanisms have been postulated. One suggests that the monitor of visual stimuli is defective and is incorrectly interpreting images. The other suggests the presence of false feedback from another visual system. In this regard, the superior colliculus, pulvinar and temporo-parietal regions may transmit signals to the monitor when the geniculocalcarine system fails. In the absence of visual input, this false internal imagery may convince the monitor or speech areas to come out with a response [9].

Although any cause of cortical blindness may potentially lead to Anton's syndrome, cerebrovascular disease is the most common [11]. In addition to the more common causes of Anton's syndrome, it has also been reported in hypertensive encephalopathy with pre-eclampsia [12], obstetric haemorrhage with hypoperfusion [13], and trauma [14], amongst others.

Our patient with bilateral occipital infarcts causing cortical blindness and visual anosognosia, fulfilled the classical description for Anton's syndrome. She maintained a fervent belief in her visual aptitude despite an obvious deficit. Her dementia was only of a mild degree and did not influence or cloud the diagnosis of Anton's syndrome.

Good recovery of visual function has been noted in conditions causing Anton's syndrome such as hypertensive encephalopathy and cortical hypoperfusion [12,13]. In these conditions, correction of the causative factor may lead to resolution of symptoms. Our patient had bilateral occipital lobe infarction, but despite a small recovery in her vision, she is unlikely to attain a substantial improvement. It would be important to consider secondary prevention, and to offer rehabilitation to such patients, should insight return.

Our case adds to the limited literature on Anton's syndrome. A suspicion of cortical blindness and Anton's syndrome should be raised in patients with atypical visual loss and evidence of occipital lobe injury.

## Consent

Written informed consent was obtained from the patient's next of kin for publication of this case report and the accompanying image. A copy of the written consent is available for review by the Editor-in-Chief of this journal.

## Competing interests

The authors declare that they have no competing interests.

## Authors' contributions

MM and SL prepared the case report and performed a literature search on Anton's syndrome. MM and BK wrote up the case report and discussion.
